# Puerperal Convulsions, and Their Prevention*Read before the Richmond Academy of Medicine and Surgery, November 24, 1896.

**Published:** 1897-02

**Authors:** John N. Upshur

**Affiliations:** Richmond, Va., Professor of the Practice of Medicine in the Medical College of Virginia, etc.


### PUERPERAL CONVULSIONS, AND THEIR PREVENTION.*

By JOHN N. UPSHUR, M.D., Richmond, Va.,

Professor of the Practice of Medicine in the Medical College of Virginia, etc.

A firm conviction of the great responsibility resting upon us in the care of the puerperal woman, and the skilful guidance through the perils of this period to its happy consummation in a safe delivery, is the motive prompting to the discussion of this condition—so frightful in its manifestations and so dire often in its consequences.

I cannot emphasize too much the importance to the patient of an early engagement of her medical attendant, that she may be closely in touch with him, and that he, by *extraordinary diligence,* may be keenly alive to every circumstance which may be to her a source of peril. Giving to his patient advice as to personal care, of diet, exercise, rest, bowels and kidneys; making regular, often frequent, analyses of the urine, to determine the presence of albumen or the lacking elimination of excrementitious solids. Often there is danger ahead when urinary analysis is negative in its results, and the nervous system is ripe for a dangerous explosion so soon as something occurs to excite the requisite reflex. I cite the following cases for sake of illustration:

CASE 1. Mrs. T., a woman of fine physique and robust health, came under my care in 1885; she would never permit me to see her until labor came on, though reported by her husband as being very dropsical. Frequent examination of her urine failed to show any albumen, though the amount of urine was scanty. Treatment, through the medium of her husband was, of course, very unsatisfactory. When labor came on, I found her the most dropsical woman I ever saw at term. She was kept under chloroform, until delivered with instruments, after a protracted labor. In four succeeding pregnancies she was never so dropsical again, but each period of pregnancy was filled with symptoms of threatened eclamp-

*Read before the Richmond Academy of Medicine and Surgery, November 24, 1896.

sia. The patient was a hearty feeder, her urine was usually scanty, albumen apparent on analysis, but not marked.

In her third pregnancy she had an attack of vertigo, followed by a period of insensibility; she has always suffered with her head. Her treatment during each pregnancy consisted of low diet, abundant drinking of lithia water, and keeping bowels in a condition of mild diarrhea. She has usually taken chloroform during the latter part of the second stage of labor, and has been safely delivered five times without a single convulsion, and is now doing well in her sixth pregnancy, having reached the end of the sixth month.

CASE 2. Mrs. W., of delicate build, in her second pregnancy, during the ninth month, had agonizing headache, which failed to respond to remedies; saw flashes of light and other objects continually before her eyes. When labor came on, she was kept under chloroform until delivered; her skin was so dry as to feel parched. Repeated examination of urine failed to detect any albumen. Within an hour after delivery, she complained of not being able to see (she had had no undue amount of hemorrhage) and began to talk wildly and incoherently. Chloroform was given to control excitement, followed by full doses of potassium bromide and pilocarpine; skin acted freely and all untoward symptoms subsided; urine showed large percentage of albumen. Convalescence uneventful until fourteenth day, when marked symptoms of septicemia developed; but she passed safely through a most dangerous illness.

CASE 3. Mrs. H. had been carefully watched during second pregnancy, urine frequently analyzed, with negative results. About the time she reached the fifth and a half month I was called to see her with a sharp attack of cholera morbus; a day or two of treatment was sufficient to restore her to health. She was discharged with caution as to imprudence in diet, and every other respect. On the evening of next day, she went out to supper, eat very heartily of almonds and raisins. I was called early the following morning to see her in what her husband called a *trance*. When I reached her she talked to me rationally, and manifested no symptoms of special gravity. At 4 p. m. she was seized with a violent puerperal convulsion, quickly followed by a second, and for a few moments I thought her dead. I bled her freely from the arm, labor was brought on, and after delivery she made an uneventful recovery—urine for the first time loaded with albumen. Her next pregnancy progressed satisfactorily to the ninth month, when she complained of distressing head symptoms, urine scanty, but free from albumen; some disturbance of vision. She was freely purged with calomel gr. vj., croton oil gtt. j, with complete relief of head symptoms. The week following, head symptoms again returned, and the dose of calomel and croton oil was repeated with as prompt relief as at first. Labor came on a few days after, and she was safely delivered, without any complication; convalescence speedy and uneventful.

CASE 4. Mrs. D., primipara, age 22, stout and plethoric. I was retained six weeks before confinement. Frequent analysis negative, except once a trace of albumen, until October 12th, when urine was found loaded with albumen, being almost solid on boiling. Was called at 10 p. m.; found she had had three convulsions, cervix rigid, dilated size of a nickel. She was bled freely from the arm, manual dilatation persisted in for five hours; child turned and delivered; a serious convulsion during the labor, which looked as though it would be fatal; no untoward symptoms during convalescence. I found that she had been complaining for a week before the labor with violent headache.

CASE 5. Was called in consultation to see Mrs. H., primipara, age 22. Saw her at 6:30 p. m. She gave the history of an unusually comfortable pregnancy. Had engaged her medical attendant *two days* before; said she had passed sufficient urine. Awoke at 5 a. m. same morning with severe headache across vault of cranium, extending over occiput and down back of neck, and some abdominal pain, which was supposed to be colic, as her time was not up (280 days) till two weeks later. Had been abstemious in her diet during lattter part of pregnancy, abstaining from eating any supper. Just before 3 o'clock p. m. she described a sensation in her head as if blood was trickling through it, and a few moments later a violent elampsic seizure developed. Her medical attendant, when he saw her in the morning, had ordered $\frac{1}{8}$ gr. doses of sulph. morphia every two hours—she having taken in all about $\frac{7}{8}$ gr. So soon as he saw her after the convulsion, he bled her freely and administered chloroform, controlling the convulsion. When I saw hers he was completely relaxed, os fully dilated and bag of waters filling the vagina—ruptured accidentally in my examination. Labor progressed satisfactorily, and she was delivered of a live baby at 8:15 p. m.—skin hot and dry, pulse soft, feeble and 120 per minute—profoundly unconscious. Another convulsion at 9 p. m. No secretion of urine since early morning; an enema of bromid. potass., sulph. morph. and camphor was administered, and she passed into a quiet natural sleep, having a good night with the exception of slight restlessness. She had also a hypodermic of 1-20 gr. sulph strychnia. On next day she had three more convulsions, but of diminishing severity and longer interval; vision impaired, and she was unable to recognize her friends before the third day; periods of consciousness alternated with periods of delirium. The baby had one convulsion on the day subsequent to its birth, but afterwards did well.

It is not my purpose to discuss the classification or causes of eclampsia; this is sufficiently done in all modern text-books. It is in the direction of such *care as will prevent the occurrence of convulsions* that I wish to consider the subject.

The cases cited as illustrative, emphasize—*first*, the necessity of early engagement, and subsequent close supervision. Case 1 shows how such care warded off trouble in five pregnancies. Case 2 points to threatened trouble indicated by the severe head symptoms with negative evidences from the urine, and, with Case 5, emphasizes this symptom and the hot, dry skin, relief coming when the skin and kidneys had their function restored. Cases 3 and 4 point to the necessity of careful dieting, and the overloading of the stomach as an exciting cause—in the one case, no albumen, and in the other albumen only twice discovered prior to the development of convulsions. Where there is excessive eating, a hypernutrition is the result in a system in which there is already a hyperplastic condition of the blood. The attempt on the part of the kidneys to eliminate increased effete matter begets kidney irritation—it fails in its function, and the appearance of albumen in the urine is the external manifestation of poisoning of the system by toxines, which, unless eliminated, result in an eclampsic explosion.

The patient's diet should be nutritious, digestible, not rich; regular exercise in the fresh air, and especial care of the bowels and kidneys.

The administration of saline cathartics should be frequent, making the mucous membrane of the bowels eliminative and derivative; an abundance of lithia water and other necessary diuretics, to stimulate the kidneys, If the patient shows evidences of anemia full doses of the tinct. chloride of iron long continued will be of value.

The treatment of the case when convulsions occur, I believe, consists imperatively in free administration of chloroform and prompt bleeding, with active purgation if the bowels are constipated; nor should we forget that the welfare of the patient depends upon as *prompt* delivering as possible—by manual dilatation of the womb and forceps or turning. When, as in Case 5, the patient remains unconscious after delivery, with symptoms of depression, as evidenced by rapid, feeble pulse, a hypodermic of strychnia nitrate will do much good by its action in sustaining the heart and diminishing cerebral congestion by its toning up effect on the vasso-motor nervous system, and at the same time sustaining the uterus in firm contraction.

I am convinced of the usefulness of morphia in conditions of rigid os and cervix in the first stage of labor—*but*, if there be symptoms of threatened eclampsia, *it is positively contraindicated, and should not be given* in the treatment of the convulsions; it arrests secretion in the skin and kidneys, and favors the retention of the effete materials in the system.

---

### THE APPENDIX AND FOREIGN BODIES.

Autopsies upon 400 persons dying of various diseases showed fecal concretions in the appendix in ten per cent., while in Renvier's collection of 454 autopsies after appendicitis there were found 179 concretions and 16 foreign bodies in this organ. Hawkins examined 60 fatal cases of appendicitis and found no foreign body whatever. Fitz analyzed 300 cases and found concretions in five per cent.—*Northwestern Lancet.*

